# CMT-3 targets different α-synuclein aggregates mitigating their toxic and inflammogenic effects

**DOI:** 10.1038/s41598-020-76927-0

**Published:** 2020-11-20

**Authors:** Florencia González-Lizárraga, Diego Ploper, César L. Ávila, Sergio B. Socías, Mauricio dos-Santos-Pereira, Belén Machín, Elaine Del-Bel, Patrick Pierre Michel, Lía I. Pietrasanta, Rita Raisman-Vozari, Rosana Chehín

**Affiliations:** 1 Instituto de Investigación en Medicina Molecular y Celular Aplicada (IMMCA) (CONICET-UNT-SIPROSA), Pasaje Dorrego 1080, 4000 San Miguel de Tucumán, Argentina; 2 Faculdade de Odontologia de Ribeirão Preto, Universidade de São Paulo, Ribeirão Preto, Brazil; 3 Departamento de Física-Instituto de Física de Buenos Aires (IFIBA, UBA-CONICET) and Centro de Microscopías Avanzadas (CMA), Facultad de Ciencias Exactas y Naturales, Universidad de Buenos Aires, C1428EHA Buenos Aires, Argentina; 4 Paris Brain Institute, Inserm U 1127, CNRS UMR 7225, Sorbonne Université UM75, Paris, France

**Keywords:** Intrinsically disordered proteins, Parkinson's disease, Biophysics, Computational biophysics, Atomic force microscopy, Confocal microscopy, Scanning electron microscopy, Transmission electron microscopy, Protein aggregation

## Abstract

Parkinson's disease (PD) is a neurodegenerative disorder for which only symptomatic treatments are available. Repurposing drugs that target α-synuclein aggregation, considered one of the main drivers of PD progression, could accelerate the development of disease-modifying therapies. In this work, we focused on chemically modified tetracycline 3 (CMT-3), a derivative with reduced antibiotic activity that crosses the blood–brain barrier and is pharmacologically safe. We found that CMT-3 inhibited α-synuclein amyloid aggregation and led to the formation of non-toxic molecular species, unlike minocycline. Furthermore, CMT-3 disassembled preformed α-synuclein amyloid fibrils into smaller fragments that were unable to seed in subsequent aggregation reactions. Most interestingly, disaggregated species were non-toxic and less inflammogenic on brain microglial cells. Finally, we modelled the interactions between CMT-3 and α-synuclein aggregates by molecular simulations. In this way, we propose a mechanism for fibril disassembly. Our results place CMT-3 as a potential disease modifier for PD and possibly other synucleinopathies.

## Introduction

Parkinson's disease (PD) is a neurodegenerative disorder related to ageing that affects 7–10 million people worldwide characterized by a progressive loss of midbrain dopaminergic neurons causing motor symptoms^1,2^. Available pharmacological interventions for PD, such as levodopa and dopamine agonists, ameliorate motor symptoms. However, these treatments lose their efficacy and cause adverse side effects^3^. In this context, there is an imperative need to develop disease-modifying therapies in order to prevent or delay disease progression.

Although the molecular basis of neurodegeneration in PD remains controversial, the central role of α-synuclein amyloid aggregation in the initiation and dissemination of the pathology seems clear^4,5^. Oligomeric α-synuclein species have been shown to elicit toxic effects by different mechanisms, such as alteration of membrane permeability with concomitant calcium influx^6^, mitochondrial damage^7^, lysosomal leakage^8^, microtubule disruption^9^, and interference with axonal transport^10^. Fibrillar species, on the other hand provoke neurotoxicity mainly by triggering inflammatory processes^11^, but also by catalyzing their own propagation^12^, destabilizing proteostasis networks^13,14^, and affecting integrity of cytosolic organelles^15^. Considering that oxidative stress and pro-inflammatory cytokines also promote the toxic aggregation of α-synuclein^16^, all these processes are suggested to integrate a vicious cycle that results in neuronal death, with subsequent spreading of toxic species into neighboring healthy neurons^17^. Thus, to efficiently modify the course of neurodegeneration in PD, an ideal drug should be capable of interfering with α-synuclein aggregation, halting the generation of toxic species, disassembling preformed toxic aggregates, and inhibiting neuroinflammatory processes. In addition, such a multi-target compound should also possess the ability to cross the blood–brain barrier (BBB), often an essential obstacle in the pharmaceutical development of medications targeting the central nervous system.

In recent years, preclinical studies suggest that tetracyclines have neuroprotective properties that might lead to their repurposing for new uses in neurodegenerative disorders, including PD^18,19^. The mechanism of action has been mainly attributed to their anti-inflammatory action^20,21^. Particularly, minocycline (MINO) has drawn a great deal of attention since this tetracycline was reported to protect from PD-neurodegeneration in preclinical models of the disease^19,22,23^. On this basis, MINO was included in a pilot clinical trial for early PD. Unfortunately, these studies revealed no beneficial effect compared to placebo, dampening hopes for therapeutic intervention of PD patients with tetracyclines^24^. Yet, a 2016 retrospective study in a Danish cohort showed that the use of a tetracycline therapy appeared to reduce the risk of PD^25^. This finding revived interest in therapeutic use of tetracyclines for PD^26^.

Like MINO, doxycycline (DOX) has also been suggested for repurposing in PD, due to the fact that it has anti-inflammatory and antioxidant properties and mitigates the loss of dopaminergic neurons in animal model^18^. In addition, DOX has been shown to inhibit the pathological aggregation of α-synuclein by reshaping toxic oligomeric species towards strains with reduced toxicity, seeding capacity and propensity to form amyloid fibrils^17^. However, the antibiotic activity of DOX represents a potential hurdle for repositioning in long-term treatments, as those required for neurodegenerative diseases. For this reason, chemically modified tetracyclines (CMTs) with diminished antimicrobial properties^26,27^ arise as attractive alternatives to DOX.

In the present study, we selected CMT-3 (also named COL-3 or Incyclinide) for further examination because it crosses the BBB, has reduced antibiotic activity and has proved to be safe in phase I clinical trials^28,29^. We demonstrated that CMT-3 was equally efficient as DOX at inhibiting α-synuclein amyloid aggregation. Furthermore, we established that MINO lacked this property. Using a combination of biophysical approaches, including electron microscopy together with infrared and fluorescence spectroscopy, we found that CMT-3 was able to bind and reshape α-synuclein oligomers into non-toxic species. Most interestingly, CMT-3 was able to disassemble α-synuclein preformed fibrils, unlike DOX and MINO, which were unable to do so. Notably, these species were unable to act as seeds in subsequent amyloid aggregation assays. They also failed to disrupt membrane integrity, induce toxicity in dopaminergic cell cultures, and were less inflammogenic in primary microglial cells. Molecular dynamics simulations revealed a plausible mechanism of interaction of CMT-3 and α-synuclein fibrils, defining the specific residues involved. Taken together, the results presented herein suggest that CMT-3 represents an ideal disease-modifying compound for repurposing in PD.

## Results

### Comparative effect of CMT-3, DOX and MINO on α-synuclein amyloid aggregation

**Figure 1:**
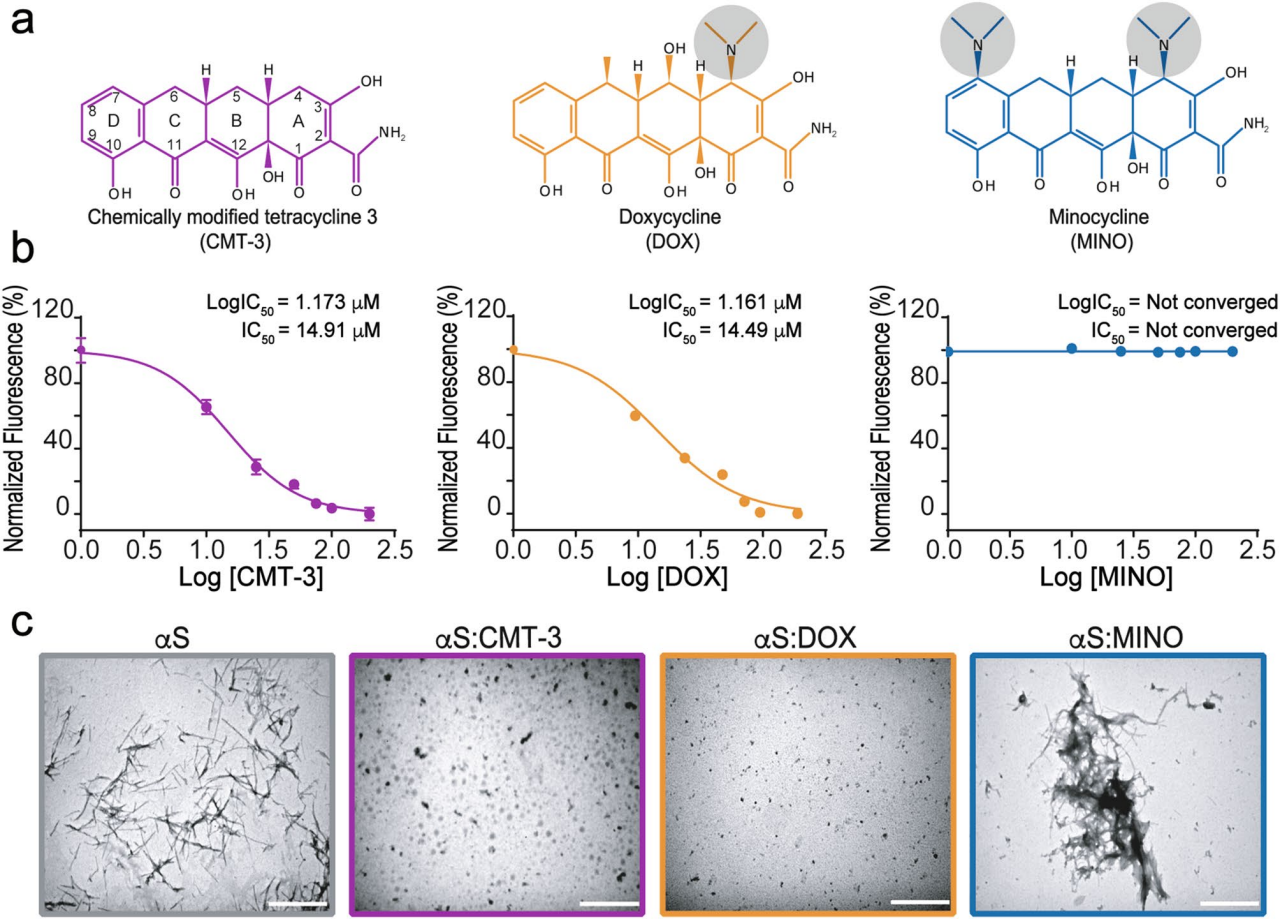
Comparative effect of CMT-3, DOX and MINO on α-synuclein amyloid aggregation. (**a**) Chemical structures of CMT-3, DOX, and MINO. Atom numbering and ring labels of the tetracycline skeleton are provided on the chemical structure of CMT-3. Note that the main difference in these structures resides in the presence or not of DMA groups designated by grey shaded circles. (**b**) Dose–response curves from endpoint ThT intensities of α-synuclein aggregation assay in the presence of different concentrations of CMT-3, DOX or MINO. IC_50_ values are mean values of three independent determinations. The data fitting is described in “Methods” section. (**c**) TEM of α-synuclein samples incubated at 37 °C for 120 h under continuous orbital agitation in the absence (αS) or in presence of CMT-3 (αS:CMT-3), DOX (αS:DOX), or MINO (αS:MINO). The scale bar corresponds to 1 μm, at ×26,000 magnification.

The chemical structures of CMT-3, DOX and MINO are shown in Fig. 1a. The decreased antimicrobial activity of CMT-3 results from the removal of the dimethyl-amino group (DMA) group in the upper peripheral region of tetracyclines, at position 4 of the A ring^30^. Nevertheless, the relevance of these substituents on the inhibition of α-synuclein aggregation is unknown. We compared the impact of CMT-3, DOX and MINO on α-synuclein amyloid aggregation by monitoring fluorescence intensity of the amyloid-specific probe Thioflavin T (ThT) (Fig. 1b). The IC_50_ values for the inhibition of α-synuclein aggregation, estimated from the dose–response curves, were 14.91 ± 2 μM and 14.49 ± 3 μM for CMT-3 and DOX, respectively. Since CMT-3 showed optimal inhibition of α-synuclein amyloid aggregation at 100 μM, hereafter all experiments were performed at this concentration. Note that MINO failed to inhibit amyloid aggregation of α-synuclein (Fig. 1b).

In order to confirm these results, we employed transmission electron microscopy (TEM) to visualize the effect of these different tetracyclines on α-synuclein aggregation. In agreement with ThT assays, while CMT-3 and DOX halted fibril growth, MINO failed to inhibit the formation of α-synuclein amyloid structures (Fig. 1c). From these results, we conclude that CMT-3 is as potent as DOX in inhibiting α-synuclein amyloid aggregation, while MINO lacks anti-aggregant properties.

### CMT-3 inhibits α-synuclein amyloid fibril formation by binding to aggregated species

**Figure 2:**
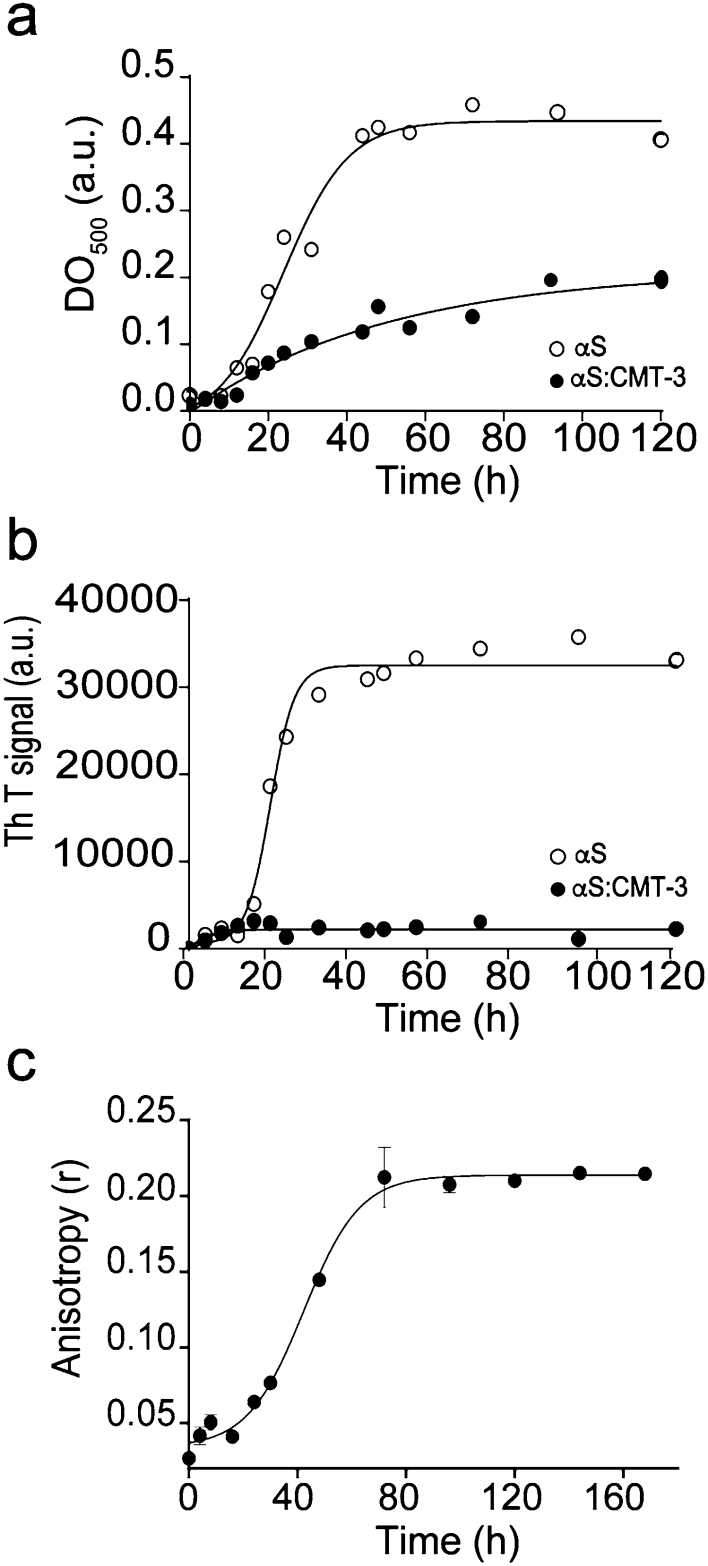
CMT-3 inhibits α-synuclein amyloid aggregation by binding to aggregated species. (**a**) Turbidimetry assay of 70-μM α-synuclein samples incubated in the absence (empty circles) or presence of 100 μM CMT-3 (full circles) and monitored at 500 nm. (**b**) ThT fluorescence of 70 μM α-synuclein samples incubated in the absence (empty circles) or presence of 100 μM CMT-3 (full circles). (**c**) Binding of CMT-3 to α-synuclein aggregated species monitored by fluorescence anisotropy. Changes in fluorescence anisotropy of CMT-3 were measured at λ_em_ 520 nm upon the addition of aliquot samples harvested at different time-points of an α-synuclein aggregation reaction. Data values represent the mean ± S.E. of 6 independent experiments.

To further characterize the effect of CMT-3 on α-synuclein aggregation, kinetic studies were performed using two complementary techniques, i.e. turbidimetry (Fig. 2a) and ThT fluorescence (Fig. 2b). While the former measures a general state of aggregation, the latter reports only the formation of amyloid aggregates. In the absence of CMT-3, both turbidimetry and ThT fluorescence techniques revealed a sigmoidal behavior for α-synuclein aggregation kinetics, with a latency time of 8 h followed by an exponential phase that reached a stationary state around 44 h, in agreement with the previous results^17^ (Fig. 2a,b). However, in the presence of CMT-3, supramolecular arrangements larger than α-synuclein monomers were formed with a lag time of 12 h (Fig. 2a). Nevertheless, aggregates did not lead to the formation of amyloid fibril structures as monitored by ThT fluorescence (Fig. 2b). This observation is in agreement with small aggregates observed in TEM studies (Fig. 1c).

Small molecules can interfere with protein aggregation by binding to different species along the aggregation pathway ^31,32^. In order to discover at which stage of the amyloid aggregation process CMT-3 binds to α-synuclein, we monitored changes in fluorescence anisotropy of CMT-3 upon addition of aliquots of an aggregation reaction harvested at different time-points. As seen in Fig. 2c, the fluorescence anisotropy (r) values of CMT-3 increased concomitantly to the appearance of aggregated species of α-synuclein (Fig. 2c). Binding data suggests that CMT-3 only interacts with aggregated forms of α-synuclein, which would therefore leave monomers available to perform their physiological functions.

### CMT-3 reshapes α-synuclein early aggregates into non-toxic species

The toxic effects of aggregated α-synuclein have been attributed to oligomeric species (αS_oli_) formed in the early stages of protein aggregation^33^. Here, we assessed the impact of CMT-3 on the structure, morphology and toxicity of these oligomeric species by infrared spectroscopy (FTIR), transmission and scanning electron microscopies (TEM and SEM), and cell culture assays.

**Figure 3:**
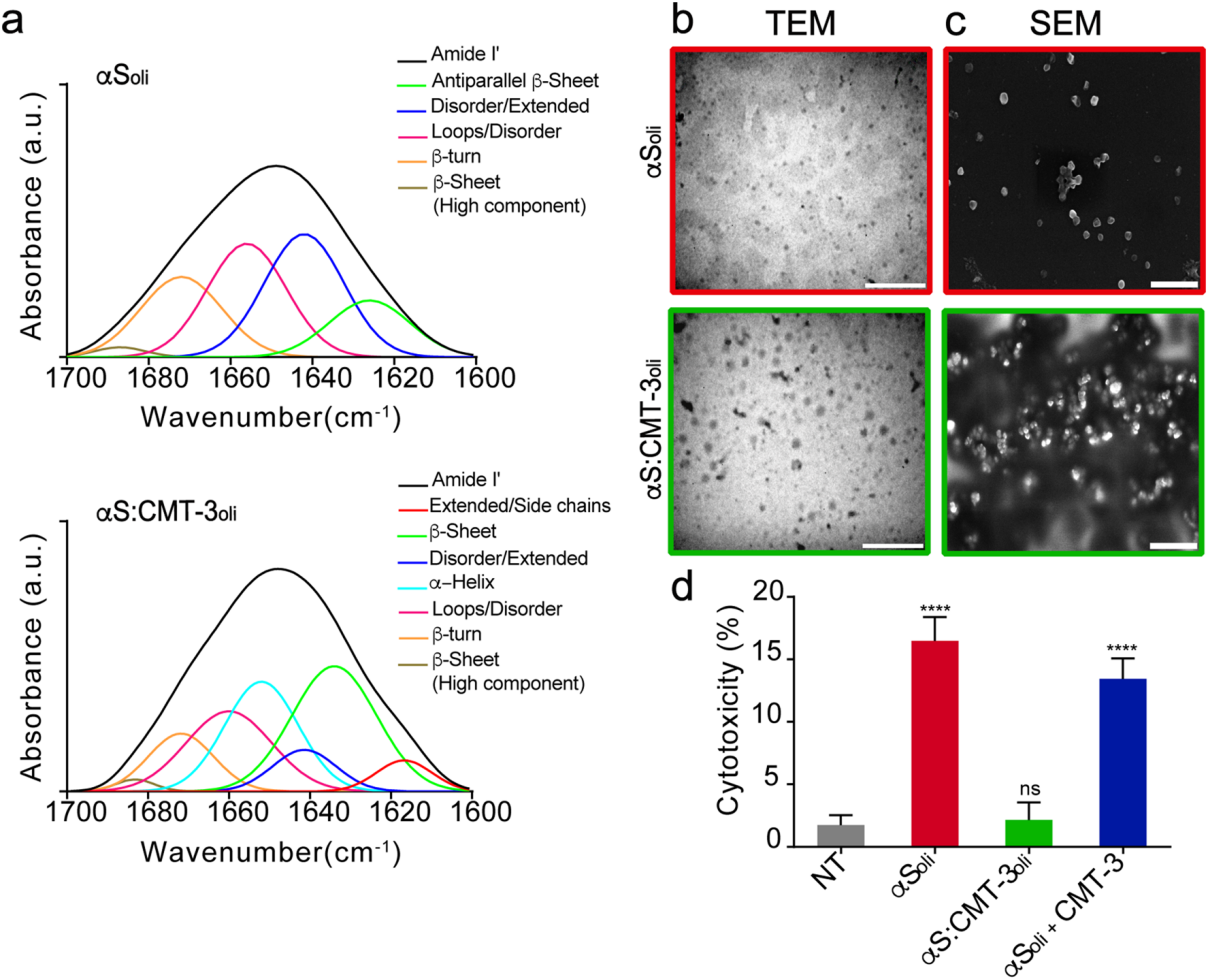
CMT-3 reshapes α-synuclein early aggregates into non-toxic species. (**a**) Analysis of α-synuclein Amide I' band after the curve fitting procedure (see “Methods”) showing the component bands: αS in the absence (top) or in the presence of CMT-3 (bottom) after 16 h incubation. (**b**) TEM and (**c**) SEM of α-synuclein samples incubated at 37 °C under orbital agitation in the absence (top) or in the presence of CMT-3 (bottom), and harvested after 16 h for observation of oligomers. The white bar corresponds to 500 nm. (**d**) LDH cytotoxicity assay in SH-SY5Y cells after the addition of α-synuclein oligomers formed after 16 h of incubation at 37 °C under orbital agitation in the absence (αS_oli_) or presence of CMT-3 (αS:CMT-3_oli_). Cytotoxicity values were normalized by the signal observed after addition of Triton X-100, which induced complete disruption of the cells. Data represents the mean ± S.E.M (n = 11). One-way ANOVA followed by Holm-Sidak’s multiple comparisons test. ****p < 0.0001 vs NT.

The Amide I' band, located in-between 1700 and 1600 cm^−1^ of the infrared spectrum, is widely used to quantify protein structural changes during the aggregation process^34,35^. This band showed significant differences between αS_oli_ samples prepared in the absence or in the presence of CMT-3 (Fig. 3a). Curve-fitting analysis was performed, as described in “Methods”, with the purpose of obtaining a relative quantification of these changes. The most relevant change observed in the presence of CMT-3 corresponded to the band located at 1642 cm^−1^, assignable to random coil regions, which presented a diminished contribution to the Amide I' from 32 to 8%. On the contrary, β-structures increased their relative contribution. In fact, antiparallel β-sheets are characterized by two bands, located at 1624 cm^−1^ and 1687 cm^−1^^36^. In the presence of CMT-3, this band shifted to 1633 cm^−1^, indicating remodelling from anti-parallel to parallel structures with an increase in their contribution from about 15 to 31.4%. CMT-3 also induced the appearance of a new band at 1652 cm^−1^, suggesting the presence of helical structures that could not be detected in the absence of the tetracycline. Another noticeable change came from comparing β-turns and open loops, which slightly decrease their contribution to Amide I' in species prepared in the presence of CMT-3 (Supplementary Table S1).

TEM images of α-synuclein samples incubated with and without CMT-3 after 16 h of orbital agitation at 37 °C revealed the formation of αS_oli_ in both samples (Fig. 3b). The average size of the resulting aggregates was calculated from the TEM images. Aggregated species of α-synuclein formed in the presence of CMT-3 were larger, with an average diameter of 70 ± 8 nm, compared to those formed without the drug, which were 30 ± 5 nm. To gain better topological and morphological information of these early aggregates, the same TEM samples were analyzed through SEM (Fig. 3c). In this way, we confirmed that the oligomers formed in the presence of CMT-3 were definitely larger in size than those formed in the absence of CMT-3.

Next, we assessed whether the morphological and structural changes in αS_oli_ induced by CMT-3 affected the toxic potential of oligomeric species. For this purpose, SH-SY5Y cells were incubated with α-synuclein oligomers prepared in the absence (αS_oli_) or in the presence of CMT-3 (αS:CMT-3_oli_) for 24 h, and cytotoxicity was measured by an LDH assay. As shown in Fig. 3d, oligomeric species obtained in our standard conditions (αS_oli_) caused a significant reduction in cell viability. In contrast, αS:CMT-3_oli_ species had no significant deleterious effect on SH-SY5Y cells. Importantly, this result was not due to a direct effect of CMT-3 per se, since CMT-3 had no protective effect when treated simultaneously with oligomers (αS_oli_ + CMT-3) (Fig. 3d). These results suggest that oligomers formed in the presence of CMT-3 (αS:CMT-3_oli_) were structurally and morphologically distinct species from αS_oli_, and furthermore were innocuous in a cell culture toxicity assay.

### CMT-3, unlike DOX, disassembles preformed α-synuclein amyloid fibrils

**Figure 4:**
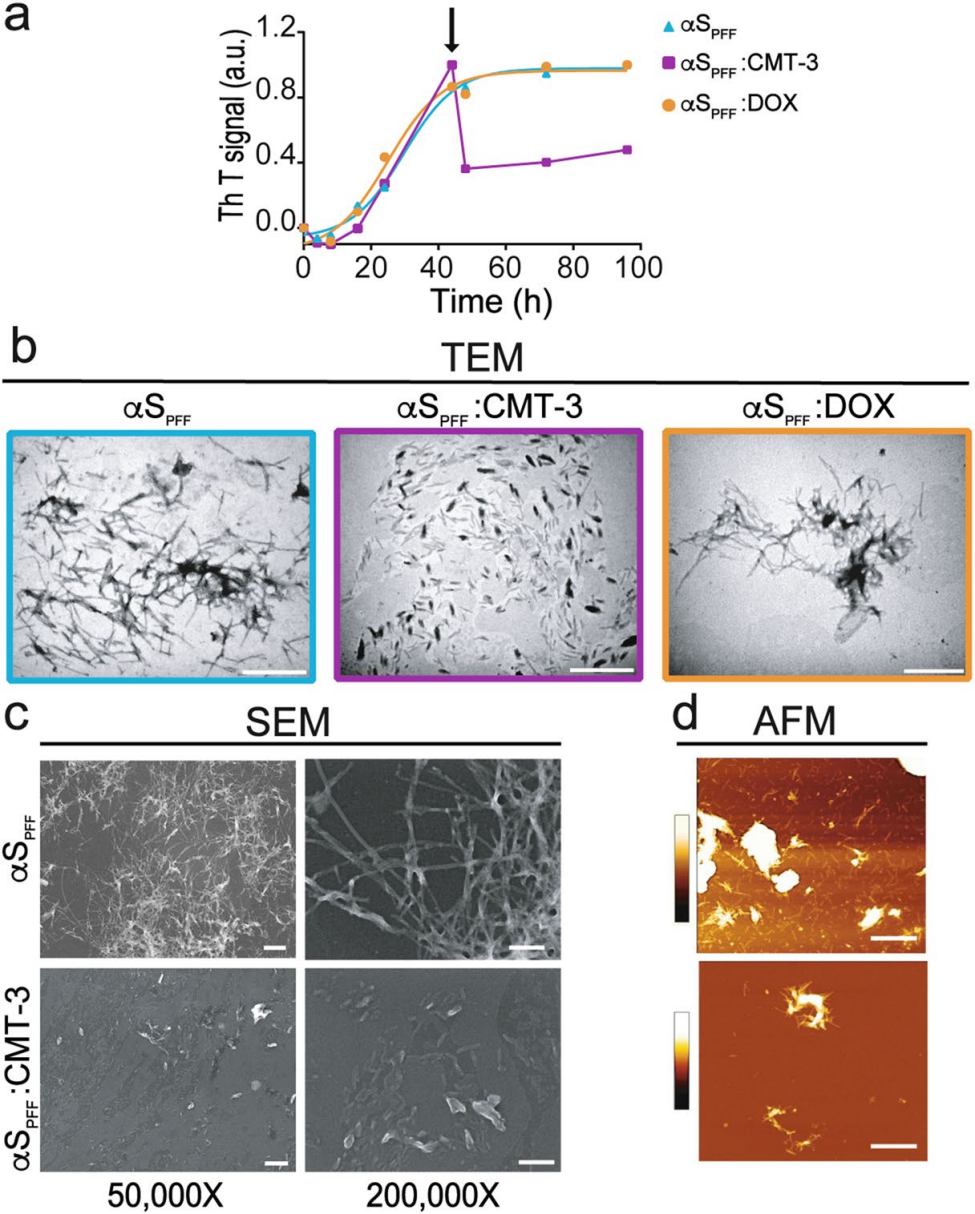
CMT-3, unlike DOX, disassembles preformed α-synuclein amyloid fibrils (αS_PFF_). (**a**) In ThT fluorescence assays, 70 μM α-synuclein was either incubated alone (blue line), or with 100 μM of CMT-3 (purple line) or DOX (orange line) added 44 h after the initiation of the aggregation process (arrow). (**b**) TEM of α-synuclein samples incubated at 37 °C under orbital agitation during 44 h to obtain fibrillar species (αS_PFF_). Samples were further incubated for another 52 h either alone or with the addition of 100 μM CMT-3 or DOX. Note the dramatic reduction in αS_PFF_ with the addition of CMT-3, but not with DOX. The scale bar corresponds to 1 μm (×26,000). (**c**, **d**) αS_PFF_ and αS_PFF_:CMT-3 species from (**b**) were further submitted to SEM and AFM. SEM scale bar corresponds to 500 nm (×50,000) and 200 nm (×200,000). AFM scale bar corresponds to 1 μm. The pseudo color scale bar represents the information of the z axis from 0 to 100 nm. The z range was chosen to allow the observation of fibrils while the aggregated fibrillar clusters are out of range since they have a height greater than 100 nm.

The fibrillary state of α-synuclein contributes to neurodegeneration through different mechanisms^11,12,14,15^. Therefore, we studied the effect of CMT-3 and DOX on disassembled α-synuclein preformed fibrils (αS_PFF_). For this, αS_PFF_ were formed by incubating α-synuclein monomers (αS_m_) for 44 h at 37 °C under continuous shaking. Subsequently, 100 μM of CMT-3 or DOX were added to these αS_PFF_ species, and incubated for an additional 52 h. As shown in Fig. 4a, incubation of αS_PFF_ with CMT-3 resulted in species, hereafter referred to as αS_PFF_:CMT-3, with a 50% decrease in ThT fluorescence intensity. On the contrary, DOX had no effect (αS_PFF_:DOX).

To confirm the capacity of CMT-3 to disassemble αS_PFF_, we used the amyloid-reporter probe Thioflavin S (ThS) and confocal microscopy imaging. In continuously shaken α-synuclein samples, the amount of fluorescent αS_PFF_ species progressively increased with time, indicating a rise in amyloid fibril formation (Supplementary Fig. S1). However, in samples where CMT-3 was added to αS_PFF_, the amount of amyloid structures within resulting species (αS_PFF_:CMT-3) diminished over time (Supplementary Fig. S1), reinforcing data described in Fig. 4a.

To acquire a deeper insight into the structure of the new species that result from the interaction of CMT-3 with αS_PFF_ (αS_PFF_:CMT-3), electron and atomic force microscopy were used. As depicted in Fig. 4b, TEM images of αS_PFF_ after 96 h of incubation with or without CMT-3 addition at 44 h, confirmed a significant reduction in both the number and size of fibrils. This property was only observed for CMT-3, as the addition of DOX to αS_PFF_ had no significant impact on fibril formation (Fig. 4b). In the absence of CMT-3, αS_PFF_ reached lengths of 640 ± 64 nm after 96 h of incubation, while αS_PFF_:CMT-3 in treated samples comprised of shorter fibrils (165 ± 38 nm) (Fig. 4b). These measurements represent the mean ± standard error for 3 independent experiments. In order to uncover a more detailed representation of the αS_PFF_:CMT-3 fragments, the same TEM samples were analyzed by SEM at different magnifications. In agreement with previous results, CMT-3-induced disruption of αS_PFF_ was evident. At 50,000×, a panoramic view of the grid confirmed that CMT-3 induced a reduction in the total amount of fibrils and aggregated fibrillar clusters (Fig. 4c). In close-up views at 200,000×, the difference in size between αS_PFF_ and αS_PFF_:CMT-3 species was observed in greater detail.

Next, atomic force microscopy (AFM) was applied in order to gain a sharper and more complete picture of the species resulting from CMT-3-induced disassembly. As shown in Fig. 4d, AFM revealed that untreated αS_PFF_ were abundant and arranged in clusters. However, αS_PFF_ treated with CMT-3 showed a clear reduction in number and length of α-synuclein aggregates and fibril clusters compared to control samples (Fig. 4d). From these results, we conclude that CMT-3, unlike DOX, was able to disrupt αS_PFF_ clusters into smaller aggregates.

### Disassembled α-synuclein PFF (αS_PFF_:CMT-3) are non-toxic and unable to either disrupt membrane integrity or promote seeding

**Figure 5:**
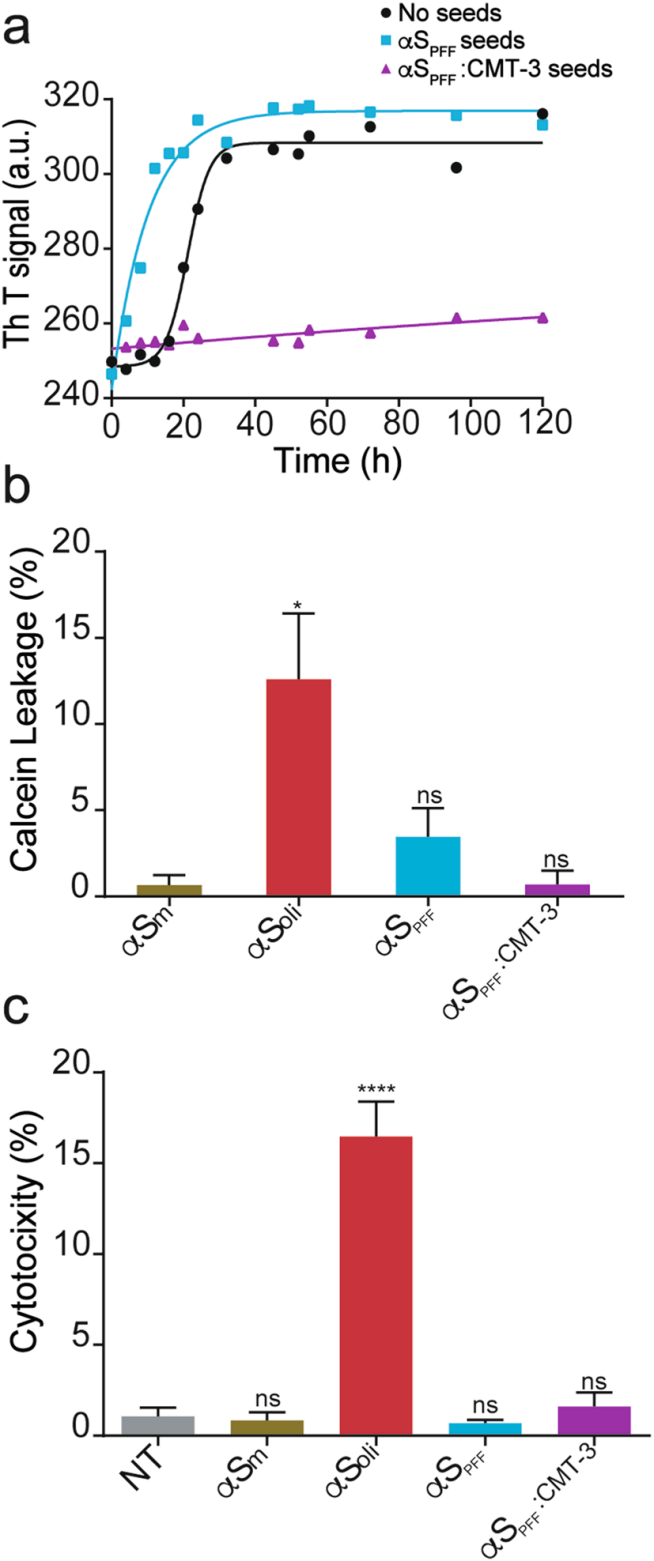
Disassembled α-synuclein PFF (αS_PFF_:CMT-3) are non-toxic and unable to either disrupt membrane integrity or promote seeding. (**a**) In seeding assays, αS_PFF_ served as efficient seeds to accelerate aggregation of α-synuclein monomers (light blue squares), while αS_PFF_:CMT-3 were not only unable to seed monomers (purple triangles), but also blocked the amyloid aggregation reaction of the monomers. The unseeded aggregation kinetics of αS_m_ (no seeds) is also shown (black circles). For this assay, solutions were incubated at 37 °C under continuous orbital agitation and aggregation was measured by ThT fluorescence emission. (**b**) Changes in liposomal membrane permeability upon the addition of α-synuclein monomers (αS_m_), α-synuclein oligomers harvested after 16 h of incubation (αS_oli_), αS_PFF_ or αS_PFF_:CMT-3. Results show that new species produced by CMT-3-driven disassembly of αS_PFF_ were unable to disrupt membrane permeability. The fluorescence signal was normalized to values obtained after addition of Triton X-100, which induced complete disruption of liposomal vesicles. (**c**) LDH cytotoxicity assay in SH-SY5Y cells after the addition of αS_m_, αS_oli_, αS_PFF_ and αS_PFF_:CMT-3. The cytotoxicity signal was normalized to values obtained after addition of Triton X-100. Data represents the mean ± S.E.M (n = 11). One-way ANOVA followed by Holm-Sidak’s multiple comparisons test. ****p < 0.0001 vs NT.

Since CMT-3 induced the disassembly of αS_PFF_ into smaller aggregated species (αS_PFF_:CMT-3), we analyzed the seeding capacity as well as the biological impact of such disrupted fibrils. To address this, we carried out seeding experiments in which αS_PFF_ and αS_PFF_:CMT-3 species were each challenged with fresh αS_m_ in order to comparatively evaluate their seeding capacity by ThT fluorescence assays. As depicted in Fig. 5a, incubation of αS_m_ in the absence of aggregate seeds presented a characteristic sigmoidal curve including the lag phase reported for amyloid aggregation kinetics. In contrast, αS_PFF_ acted as seeds for the reaction, abolishing the lag phase and triggering rapid aggregation of αS_m_ into amyloid fibrils, in agreement with previous reports^37^. However, αS_PFF_:CMT-3 species were unable to elicit this type of response, indicating their inability to act as polymerization seeds. In addition, these data suggest that CMT-3 not only disassembles amyloid fibrils, but also might remodel the resulting amyloid fragments (Fig. 5a). Interestingly, αS_PFF_:CMT-3 species even hindered the aggregation process of native αS_m_, implying that amyloid fibrils disassembled by CMT-3 could per se act as anti-aggregant agents.

To test the impact of αS_PFF_:CMT-3 species on membrane integrity, content leakage assays with synthetic membranes were performed. For this, we monitored the release of calcein entrapped in DOPC:DOPA (1:1) lipid vesicles upon the addition of αS_m_, αS_oli,_ αS_PFF_ and αS_PFF_:CMT-3. As shown in Fig. 5b, monomeric species and αS_PFF_ did not induce significant changes in membrane permeability. On the contrary, αS_oli_ induced an increase in the release of the fluorescent probe from liposomes. Interestingly, αS_PFF_:CMT-3 species were incapable of altering membrane permeability (Fig. 5b).

In order to determine if αS_PFF_:CMT-3 species were neurotoxic we measured the release of cytosolic enzyme lactate dehydrogenase (LDH) in SH-SY5Y human neuroblastoma cells, an in vitro model of dopaminergic neurons. In accordance with membrane permeability assays, αS_oli_ triggered cytotoxicity while αS_PFF_ did not, as previously reported^38^. Consistent with membrane permeability assays, αS_PFF_:CMT-3 species failed to induce LDH release in the culture medium (Fig. 5c). The fact that αS_PFF_:CMT-3 species were unable to act as polymerization seeds, alter lipid membrane permeability, nor exhibit toxicity in a cell culture model of dopaminergic neurons, has important implications from a therapeutic perspective.

### Disassembled α-synuclein PFF (αS_PFF_:CMT-3) are less inflammogenic for microglial cells

Fibrillary aggregates of α-synuclein possess the capacity of triggering brain neuroinflammation, a process thought to constitute a core component of PD^11,39^. For this reason, we compared the impact of 24 h-treatments with αS_PFF_ or αS_PFF_:CMT-3 on microglial cell activation by measuring levels of TNF-α a pro-inflammatory cytokine, and glutamate, a non-cytokine inflammation marker^40,41^. Considering the reported anti-inflammatory effects of CMT-3^27^, we used αS_PFF_:CMT-3 samples that were dialyzed prior to addition to microglial cell cultures to remove the unbound fraction of tetracycline (see “Methods” for details).

**Figure 6:**
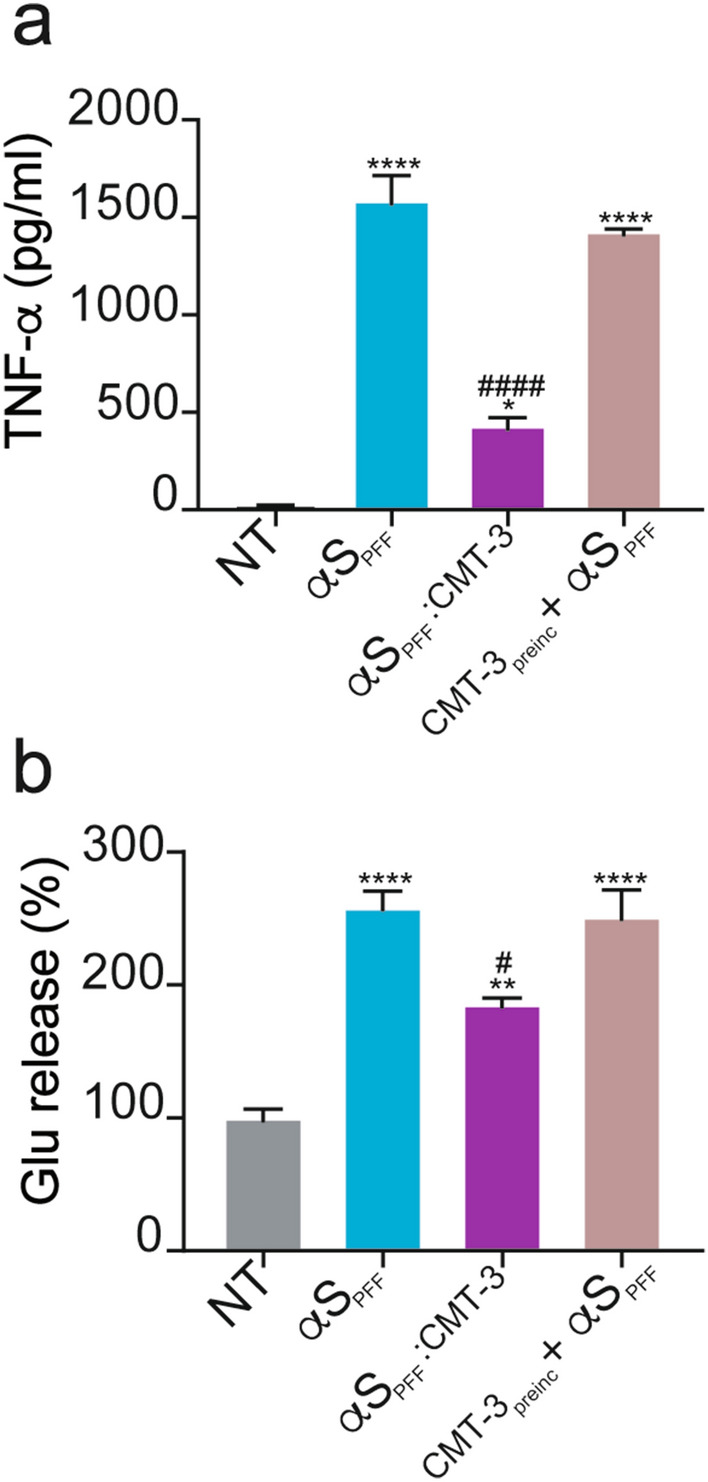
Disassembled α-synuclein PFF (αS_PFF_:CMT-3) are less inflammogenic for microglial cells. (**a**) TNF-α release in microglial cells treated for 24 h with αS_PFF_, αS_PFF_:CMT-3 (70 μg/ml), or intact αS_PFF_ in wells pre-incubated with CMT-3 (CMT-3_preinc_ + αS_PFF_). (**b**) Quantification of glutamate release after exposure of microglial cultures to the same treatments as in (a). Data represents the mean ± S.E.M (n = 11). One-way ANOVA followed by Holm-Sidak’s multiple comparisons test. *p < 0.05, **p < 0.01, ****p < 0.0001 *vs* NT. #p < 0.05, #### p < 0.0001 vs αS_PFF_._._

In accordance with our previous reports^11,42^, treating microglial cells with 70 μg/ml of αS_PFF_, a concentration known to induce activation in this model system, robustly stimulated TNF-α release (Fig. 6a). The inflammatory potential of αS_PFF_:CMT-3 species appeared, however, significantly reduced compared to intact αS_PFF_ (Fig. 6a). Considering its known anti-inflammatory effects^27^, unbound CMT-3 was removed by dialysis before treatment (described in “Methods”). Nevertheless, the anti-inflammatory properties of CMT-3 were not responsible for the difference observed, since microglia cell cultures pre-incubated with CMT-3 and then treated with αS_PFF_ (CMT-3_preinc_ + αS_PFF_) showed no statistical differences in TNF-α release compared to αS_PFF_ (Fig. 6a).

Following, glutamate release was measured as an alternative inflammation marker for microglial cells (Fig. 6b). As expected, a treatment of microglial cells with αS_PFF_ substantially increased the release of the neurotransmitter^11^, while αS_PFF_:CMT-3 produced an attenuated response compared to that of intact αS_PFF_, (Fig. 6b). Again, cells pre-incubated with CMT-3 and later treated with αS_PFF_ (CMT-3_preinc_ + αS_PFF_) showed no difference with respect to αS_PFF_-treated conditions. Monomeric forms of α-synuclein, in contrast to αS_PFF,_ had no or limited influence on the production of these inflammation markers (Supplementary Fig. S2).

In summary, molecular species resulting from the disassembly of αS_PFF_ by CMT-3, were less effective at activating microglia than intact αS_PFF_, and this ability was unrelated to intrinsic anti-inflammatory properties of this tetracycline.

### Binding mode of CMT-3 to α-synuclein fibrils and subsequent disassembly mechanism

**Figure 7:**
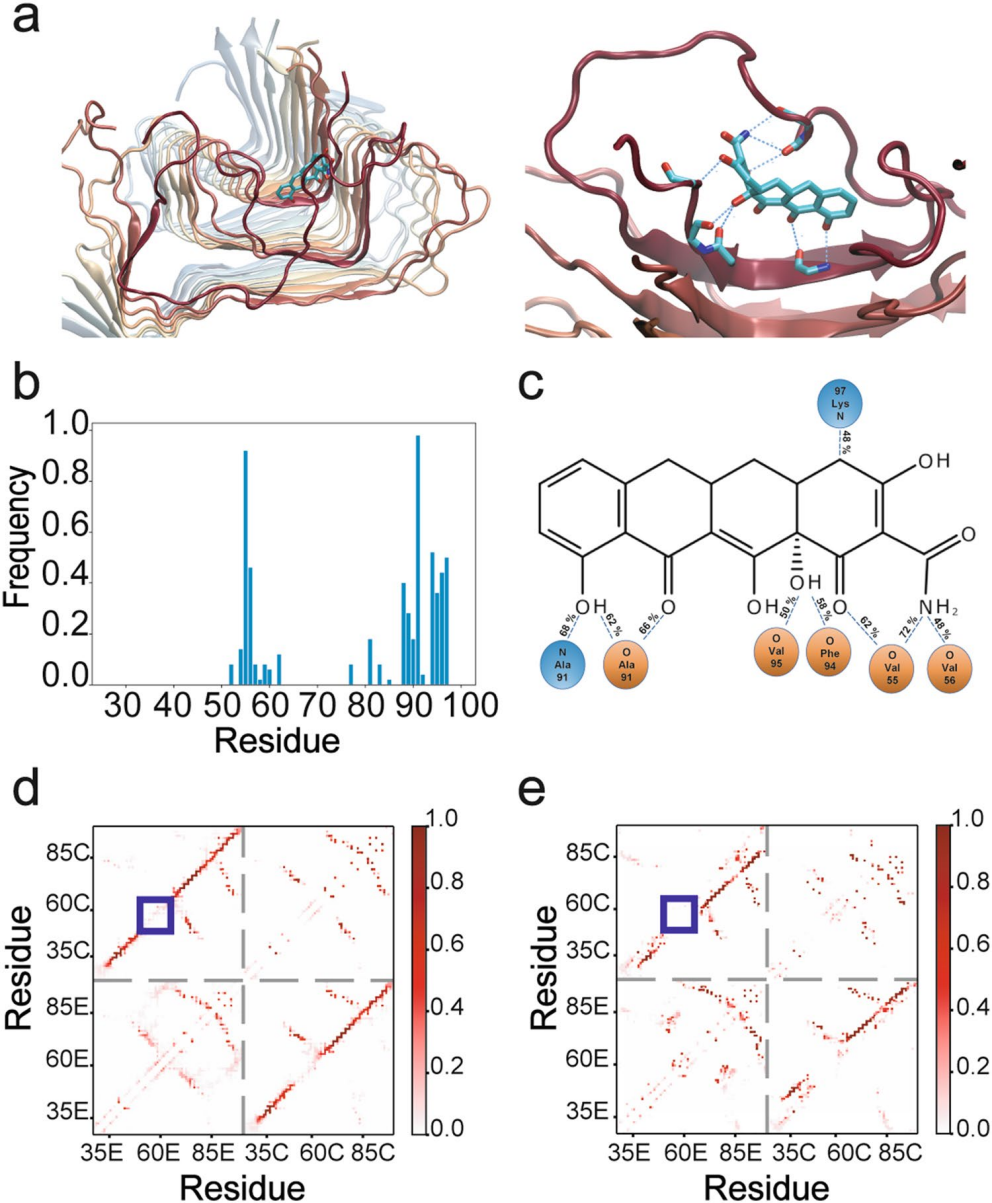
Binding mode of CMT-3 to α-synuclein fibrils and subsequent disassembly mechanism (**a**) Side and close-up views of the putative complex formed between CMT-3 and α-synuclein fibrils. The protein backbone is represented in cartoon while the ligand is depicted in ball-and-stick representation. The hydrogen bond network between the protein and the ligand is represented as a discontinued blue line in the enlarged side view. (**b**) Contact frequency between CMT-3 and α-synuclein amino acid residues. (**c**) Schematic representation of the hydrogen bonds between CMT-3 and atoms from amide and carbonyl groups in the main chain of α-synuclein. Contact map between the residues at the edge (E) or the core (C) of the α-synuclein fiber in the absence (**d**) or presence of CMT-3 (**e**). The color bar scale represents the frequency contact along the simulation time.

In order to shed light on the binding mode of CMT-3 to aggregated α-synuclein, we performed high throughput unbiased molecular dynamic simulations. Briefly, in these simulations the ligand was randomly placed in a water box around the protein and the binding events were analyzed using the Markov state model framework^43^. This framework allows the identification of long timescale phenomena by performing multiple shorter simulations in parallel. In this way, five binding poses were identified with the first binding pose having the largest implied time scale (Supplementary Fig. S3). As seen in Fig. 7a, this binding pose involves the interaction of CMT-3 with the folded monomer at the edge of the α-synuclein fibril and perpendicular to the fibril elongation axis. An analysis of the contact frequency between the protein and the ligand revealed that CMT-3 interacts mainly with residues Ala91 (98%) and Val55 (92%), followed by Phe94 (52%), Lys97 (50%), Ala56 (46%) and Lys96 (44%) (Fig. 7b). As we suggested previously^26^, the interaction occurred mainly between carbonyl and amide atoms of the protein main chain and the hydrogen bond donor and acceptor groups present in the lower peripheral region of the tetracycline core structure (Fig. 7c). The interaction is further stabilized by hydrophobic contacts between the side chains of Val55, Ile88 and Phe94 and the carbocyclic rings in CMT-3. In this way, CMT-3 would interfere with the fibril’s capacity to recruit new monomers by blocking the residues with capability to form cross-β hydrogen bonds at the edge of the protein scaffold. Moreover, CMT-3 seems to disrupt the native contacts present in the fibrillary structure.

Upon analysis of the contact map in the solid-state NMR (ssNMR) structure deposited in the Protein Databank, interchain contacts between residues in neighboring monomers were observed as an offset diagonal in the contact matrix (Supplementary Fig. S4). These lines in the contact map correspond to in-register parallel cross-β sheets. It should be pointed out that this contact map corresponds to the idealized structure deposited in the database. On the other hand, the dynamic picture gained from these simulations shows that while the contacts in the hydrophobic core of the Greek key motif of the fibril are quite conserved (residues 46–54 and 63–96), there are certain regions of the structure with increased flexibility (residues 1–45, 55–62 and 97–120). These flexible segments can be seen as regions with a large fluctuation over their average position in the structure (see Root mean square fluctuation (RMSF) plot in Supplementary Fig. S5), or alternatively, as contacts with lower frequency on the contact map (marked with a blue square in Fig. 7d). These observations are in agreement with experimental data showing that the loop in the segment 51–67 is more disordered^44^. In our simulation studies, we found that upon binding of CMT-3 to residues 91–95, the flexible region comprised between residues 55–62 detaches from the protein scaffold, as revealed by the disappearance of the native contact on the contact map (Fig. 7e). Instead, this flexible region folds back into CMT-3 keeping a strong interaction between CMT-3 and the backbone atoms of Val55 and Ala56. We hypothesize that this could represent an initial step in fibril disassembly, which could then progress upon binding of additional CMT-3 molecules into adjacent regions.

## Discussion

Despite the enormous efforts poured into developing treatments for Parkinson’s disease (PD), a definitive solution is yet to be found. Several promising molecules that target PD pathogenic pathways such as α-synuclein aggregation, oxidative stress, and neuroinflammation, had limited success in clinical trials^45^. Due to the multifactorial basis of the disease, a multitarget drug with efficient activity against all these processes could open new opportunities for effective treatments.

We selected CMT-3 (also named COL-3 or Incyclinide), because of its close structural analogy to DOX and MINO, two other tetracycline derivatives reported to exert neuroprotective effects in PD models. In addition, CMT-3 is a tetracycline derivative with reduced antibiotic activity that is ready to enter in phase II clinical trials. Here, we show that CMT-3 inhibited α-synuclein amyloid aggregation in a concentration dependent manner as efficiently as DOX^17^, remodeled α-synuclein oligomers into non-toxic species, and even disaggregated preformed α-synuclein fibrils (αS_PFF_). On the contrary, DOX did not induce any changes in αS_PFF_ structures. Moreover, we unveiled that MINO, also failed to inhibit α-synuclein amyloid fibril formation. Importantly, α-synuclein species disaggregated by CMT-3 treatment (αS_PFF_:CMT-3) were non-toxic, less inflammogenic and did not serve as seeds for further amyloid aggregation. Considering that α-synuclein aggregates have been shown to spread from the brain of patients developing PD to grafted neurons^46^, loss of seeding capacity might be therapeutically relevant. Furthermore, disaggregated fragments (αS_PFF_:CMT-3) inhibited the protein aggregation reaction, acting as anti-aggregants themselves. Other compounds have been shown to disassemble α-synuclein amyloid fibrils, including epigallocatechin gallate (EGCG), baicalein, rifampicin and oleuropein derivatives^47–50^. However, to our knowledge this is the first study to evaluate the inflammogenic potential of the disassembled species. A main difference between CMT-3 and other amyloid-disrupting compounds is that this tetracycline did not bind to monomeric α-synuclein, and it would not interfere with the monomer’s physiological functions^51^. These attributes, combined with the relative innocuousness, weakened antibacterial activity^27,52^, ability to cross the BBB, antioxidant and anti-inflammogenic properties^28,29^, render CMT-3 as an attractive candidate for repurposing in PD and other synucleopathies.

Tetracyclines, mainly DOX and MINO, have a number of neuroprotective properties in different PD animal models in which neurodegeneration and/or inflammation are induced by 6-hydroxydopamine (6-OHDA), 1-methyl-4-phenyl-1,2,3,6-tetrahydropyridine (MPTP), Paraquat or lipopolysaccharide^18–20,53,54^. Promising results, mainly attributed to anti-inflammatory and antioxidant capabilities, prompted a clinical trial to test the efficacy of MINO against early PD in 2008. Unfortunately, MINO failed to show any beneficial effects in this study. This trial seemed to bury the hopes of repurposing tetracyclines for PD. However, it is widely accepted that toxic amyloid aggregation of α-synuclein is the key molecular event that provokes the vicious cycle of aggregation, neuroinflammation, and other pathological hallmarks in PD. Here, we demonstrated that MINO was incapable of inhibiting this toxic α-synuclein aggregation in vitro, which may possibly explain retrospectively the absence of clinical improvement in PD patients. On the contrary, DOX and CMT-3 did efficiently prevent formation of toxic α-synuclein amyloid aggregates in vitro, with the latter even possessing ability to disaggregate α-synuclein amyloid fibrils. This implies that structural differences in tetracyclines conceal properties that justify re-examining their therapeutic potential for PD.

CMT-3, like many other tetracyclines, has a potent inhibitory effect on the activity, activation, and production of matrix metalloproteinases (MMP)^55,56^. For this reason, it has been developed as an anti-metastatic and has been tested in clinical settings, especially for advanced soft tissue and AIDS-related Kaposi sarcomas, and high-grade gliomas^57,58^. CMT-3 has also been shown to decrease lipopolysaccharide-induced microglial activation and cytokine release in the brain, which has been attributed to an inhibitory effect on protein translation^27^. Recently, a molecular mechanism has been provided for the effect of tetracyclines (including CMT-3) at attenuating eukaryotic protein synthesis^59^. This effect also seems to be responsible for enhancing longevity and proteostasis in a *C. elegans-*ageing model^59–61^. Therefore, while the effects we describe for CMT-3 on α-synuclein amyloid aggregation result purely from ex vivo and in vitro assays, this additional mechanism could indirectly help prevent protein aggregation in vivo. Ideally, CMT-3 could attack amyloid aggregation on many fronts by tempering translation, inhibiting aggregation of what has already been translated; disrupting what has already been aggregated and reducing previously triggered inflammation and oxidation.

Considering the pleiotropic nature of tetracyclines, and the fact that chemical modification of substituents on the ring skeleton can bestow different properties, results presented herein highlight how DMA groups that endow antimicrobial activity^30^, interfere with the anti-aggregant properties of the molecule. Specifically, the DMA present in MINO and absent in both DOX and CMT-3 (on C-7) seemed to obstruct the ability of the tetracycline to inhibit α-synuclein amyloid aggregation, while the DMA present in MINO and DOX (on C-4), prevented the ability to disassemble αS_PFF_. This suggests that substituents on the upper peripheral region of tetracyclines could influence the anti-aggregant and disassembling properties of these molecules, a structure–activity insight that may represent precious information for future drug design.

A sharper picture of the interaction between CMT-3 and αS_PFF_ was gained through molecular modelling. Although existing computational power limits the simulations to only the first steps of this process, we think that a model based on high-throughput molecular dynamic simulations can provide a hint on the mechanism of ligand binding and fibril disassembly. According to our model, CMT-3 would prevent aggregation by blocking specific amino acid interaction in the aggregation-prone segment A^88^AATGFVK^96^ in α-synuclein, in a mechanism similar to the one proposed for EGCG^62^. In particular, the presence of hydrogen bond donors and acceptors in the lower peripheral region of CMT-3 seems to be critical for the interaction with carbonyl and amide groups present in the main chain of the protein, impairing its ability to recruit subsequent α-synuclein monomers. Also, the hydrophobic nature of the cyclic rings in CMT-3 would serve to target the hydrophobic core of the fibril. The binding mode is similar to the one recently proposed for the binding of DOX to Aβ amyloid fibers^63^. In this way, the binding mode seems to be dependent on the conformation of the aggregation-prone segment rather than on its sequence, and would explain the capability of tetracyclines to inhibit the amyloid aggregation of diverse proteins such as Aβ^64^, PrP^65^, α-synuclein^17,66^ or β2-microglobulin^67^. Furthermore, we propose that CMT-3 might act as a molecular zipper, with the flexible loop folding back into the aggregation-prone segment, disrupting the Greek key motif and initiating fibril disassembly. It must be stressed that based on current simulations, we are not able to explain the different capabilities of CMT-3, DOX and MINO, since the basic structural motif is shared among all of them. Nevertheless, we hypothesize that the DMA groups on the upper peripheral region might play a role by introducing steric impediments that would affect the molecule’s anti-aggregant activity. Further studies are needed to validate this hypothesis.

Considering the feasibility of a CMT-3 based treatment, long-term therapy with antibiotic-related compounds could impose a selective pressure and therefore increase the risk of microbial resistance development and native microbiota disturbance. However, several clinical studies indicate that at low doses, below the minimal inhibitory concentration (MIC), antibiotic therapy is safe, does not kill the native microbial communities nor perturb the microbia/human interaction even after following years of treatment^26,68–70^. In addition, previous reports showed that the antimicrobial action of CMT-3 was dramatically reduced compared to that of doxycycline^27^. Therefore, considering its very low antimicrobial activity and the concentration required for its neuroprotective action, long-term therapy with CMT-3 should not negatively affect human native microbiota. Nevertheless, more experimental evidence is required to support this hypothesis.

Whether CMT-3 administration is able to prevent α-synuclein deposition into Lewy bodies or even disassemble α-synuclein fibrils in vivo*,* either in α-synuclein overexpressing PD mice models or in animals injected with αS_PFF_ is unknown, and remains beyond the scope of this report. Also undetermined are the effects of CMT-3 on different strains of αS_PFF_ or those that harbor mutations that predispose to PD. Although the data that support repositioning tetracycline derivatives for PD stem from either in vitro or toxin-based rodent models, a recent large retrospective study revealed that tetracycline therapy is associated with reduced risk of PD^25^, hinting that there is still as much to be learned from these compounds as there is hope for their repurposing in neurodegenerative diseases.

In conclusion, although a number of compounds have been shown to inhibit α-synuclein amyloid aggregation, few are already being tested in clinical trials, traverse the BBB, and disrupt mature αS_PFF_ like CMT-3. These properties add to the previously known anti-inflammatory and antioxidant abilities of tetracyclines. Therefore, CMT-3 represents an ideal multi-target compound to be considered for clinical studies and potential repurposing in PD.

## Methods

### Preparation of α-synuclein

Recombinant wild-type human α-synuclein was expressed in *Escherichia coli* using plasmid pT7-7 encoding for the protein sequence. Purification was performed as previously described^71^. Protein purity was assessed by electrophoresis in polyacrylamide gels under denaturing conditions (SDS-PAGE). The stock solution of α-synuclein was prepared in 20 mM HEPES, 150 mM NaCl, pH 7.4. Prior to aggregation assay, the protein stock solutions were centrifuged for 30 min at 12,000 × *g* to remove microaggregates. Protein concentration was determined by the measurement of absorbance at 280 nm using extinction coefficient ε_275_ = 5600 cm^−1^ M^−1^.

### Protein aggregation assays

The aggregation protocol was adapted from previous studies^72^. The different aggregated species were formed by incubating recombinant α-synuclein samples (70 μM) in 20 mM HEPES, 150 mM NaCl, pH 7.4, in a Thermomixer Comfort (Eppendorf) at 37 °C under orbital agitation at 600 rpm in the absence or in the presence of CMT-3, DOX and MINO.

### Thioflavin T assay

Aggregation studies of α-synuclein in the absence or in the presence of CMT-3, DOX and MINO were performed by measuring the fluorescence emission of ThT at different time-points according to LeVine^73^. Changes in the emission fluorescence spectra with the excitation wavelength set at 450 nm was monitored using an ISS (Champaign, IL) PC1 spectrofluorometer. The dose–response curves for the aggregation inhibitory effect was fitted to the following equation:

$$I_{F} = \frac{100}{{1 + 10^{{(\left[ {Ligand} \right] - \log IC_{50} )}} }},$$

where *I*_*F*_ is the normalized fluorescence intensity, [Ligand] is the tetracycline concentration, and IC_50_ is the concentration at which aggregation is inhibited at a 50%.

### Turbidity measurements

Turbidity caused by α-synuclein aggregation in the presence or in absence of CMT-3 was examined with absorbance at 500 nm. The absorbance of each solution was monitored in a Beckman DU 7500 spectrophotometer.

### Transmission electron microscopy (TEM) and scanning electron microscopy (SEM)

Samples (50 μl) of a 70 μM α-synuclein solution were adsorbed onto glow-discharged 200 mesh carbon film coated copper grids (Electron Microscopy Sciences) and stained with uranyl acetate (2%). Excess liquid was removed and grids were allowed to air dry. The grids prepared in this way were analyzed using TEM and SEM. For the former, images were captured using a Philips 301 transmission electron microscope, while for SEM, the images were taken at 3 kV with a working distance between 3 and 3.8 mm and high vacuum in a Zeiss Supra FE-SEM microscope. In order to assess the results in an unbiased manner, 30 representative fields were selected from each grid.

### Atomic force microscopy (AFM)

Aliquots (10 μL) of amyloid samples were deposited on the surface of freshly cleaved mica, washed three times with Milli-Q water, and dried with dry nitrogen. AFM images were acquired with a Multimode 8 AFM (NanoScope V Controller, Bruker, Santa Barbara, CA, USA). Tapping mode imaging was carried out in a dry nitrogen atmosphere using silicon rectangular cantilevers with rotated pyramidal tips. The etched silicon probe with rotated tip (RTESP) probe (Bruker) specifications include a nominal tip radius of 8 nm curvature, nominal cantilever spring constant k of 40 N/m, and nominal resonant frequency of 300 kHz. The AFM images were processed using commercial NanoScope Analysis software (Bruker) to remove the background slope.

### Fluorescence confocal microscopy

α-Synuclein amyloid fibrils formed at different time-points were centrifuged 30 min at 12,000 × *g*. The pellets were resuspended and incubated with 0.2 mg/ml Thioflavin S for 2 h at room temperature under stirring. Then, fibrils were washed by centrifugation and resuspended in freshly prepared buffer three times to remove excess of Thioflavin S. The fibrils obtained were finally resuspended in buffer 20 mM HEPES, pH 7.4, to be observed under a Zeiss LSM 800 confocal microscope (Germany).

### Fluorescence anisotropy

The interaction between α-synuclein aggregates and CMT-3 was evaluated by measuring the fluorescence anisotropy of the intrinsic fluorescence of CMT-3. Briefly, aliquots (100 μl) were taken at different times of the α-synuclein aggregation reaction, CMT-3 was added, and then fluorescence anisotropy was measured. The fluorescence anisotropy was determined in an ISS PC1 photon counting spectrofluorometer with thermostated cuvette support, and equipped with polarizers for both the excitation beam and the emission beam, adjusting the excitation and emission wavelengths at 460 nm and 520 nm, respectively. The fluorescence anisotropy was calculated according to:

$$rss \, = \, \left( {I_{VV} {-} \, g \, l_{VH} } \right)/\left( {lvv \, + \, 2 \, g \, l_{VH} } \right),$$

where *g* is an instrumental correction factor that normalizes the sensitivity of the photomultiplier, while *I*_*VV*_ and *l*_*VH*_ are the intensities of the fluorescence with the emission polarizer in parallel or perpendicular, respectively to the excitation polarizer.

### Fourier transformed infrared spectroscopy

α-Synuclein for infrared studies (280 μM) was prepared in 20 mM HEPES, 150 mM NaCl, pD 7.40. Then, 400 μM of CMT-3, also in the same buffer, was added and the mixture incubated for 16 h under orbital agitation at 37 °C. To avoid αS_m_ contribution, an Amicon Ultra-0.5 100 kDa cut-off filter was used recovering about 50 μl from the upper side of the filtration system. Each sample was assembled in a liquid cell (Harricks Scientific, Ossioning, NY) between two CaF_2_ windows with a path length of 50 nm. The sample chamber was constantly purged with dry air. The spectra were obtained by averaging 256 interferograms collected with a nominal resolution of 2 cm^−1^ and apodized with a Happ-Genzel function in a Nicolet 5700 spectrometer equipped with a DTGS detector (Thermo Nicolet, Madison, WI) as previously described^74^. Quantitative information on protein structure was obtained through decomposition of the Amide I' band into its constituents as previously described^75,76^. Structural analyses, either in the absence or in the presence of CMT-3 were repeated three times to test the reproducibility of the measurements. In each case, the differences among the experiments was around 3%^77^.

### Lipid membrane permeability

Calcein-loaded LUVs of PA/PC (molar ratio of 1:1) were prepared by hydrating lipid films in the presence of calcein and separated from free dye using a Sephadex G-75 column (Sigma). Different aggregates of α-synuclein (140 μM) were added to 50 μM of lipid vesicles. During incubation, changes in the fluorescence intensity of the different samples were monitored at λ_exc_ = 490 nm and λ_em_ = 510 nm in a ISS PC1 spectrofluorometer (Champaign, IL, USA). Total dye release was completed by the addition of 0.2 vol% Triton X-100. The percentage of probe release was calculated as follows:

$$\% {\text{Dye release }} = \left( {I_{{\text{F}}} - I_{{\text{B}}} } \right) \times {1}00/\left( {I_{{\text{T}}} - I_{{\text{B}}} } \right),$$

where *I*_F_, *I*_T_, and *I*_B_ are the fluorescence intensity of the dye released by the protein, total dye released, and control blank, respectively.

### Dialysis

Samples tested in cell cultures were dialyzed twice against HEPES 20 mM, NaCl 150 mM, p.H. 7.4 for 24 h using Slide-A-Lyzer 10 k Dialysis Cassettes (Thermo Fisher). Removal of CMT-3 from the samples was confirmed by spectroscopic methods.

### Lactate dehydrogenase (LDH) release assay

SH-SY5Y cells were grown in DMEM supplemented with 10% fetal bovine serum (FBS) and 1% penicillin/streptomycin (PS), at 37 °C and 5% CO_2_. 14 μM of different samples (αS_oli_, αS:CMT-3_oli_, αS_oli_ + CMT-3, αS_PFF_, αS_PFF_:CMT-3) were added to SH-SY5Y cells (15,000 cells/well) in a 96 well plate. After 24 h of incubation, LDH release assay was performed using LDH kit (Roche) according to the manufacturer’s instructions. The absorption values at 490 nm and 692 nm were determined using a TECAN microplate reader. The percentage of LDH release was plotted as the difference in the absorbance value of 490 nm and 692 nm. All experiments were performed in sextuplicate, considering 100% release for TritonX-100 treated cells.

### Microglial cell isolation and seeding

Coating procedure was performed as described previously^11,42^. Briefly, polycation coating solutions containing 1 mg/ml polyethyleimine (PEI) were applied to culture vessels for 2 h at 37 °C, then washed with PBS and used for cell seeding. Newborn mice were sacrificed and had their brain rapidly dissected. Two mouse brains were plated onto PEI-coated Corning T-75 flasks (Sigma-Aldrich) with 12 ml of complete medium (DMEM + 10% FBS and antibiotics) and incubated at 37 °C in an atmosphere of 95% air and 5% CO_2_. 10 ml of culture medium were removed 48 h after plating to eliminate floating debris and 10 ml of fresh medium was added. No additional medium was added until the total disappearance of other cell types. To produce subcultures, microglial cells were recovered by trypsin proteolysis using an EDTA (2 mM)-trypsin (0.05%) solution and seeded onto no-coated Nunc 96-well plates at density of 20,000 cells per well for 24 h using the N5B medium (N5 medium + 5% HS + 0.5% FBS + 5 µM glucose) added with glycine 100 µM.

### Primary microglial cell treatments

Primary microglial cells were treated with αS_PFF_ or αS_PFF_:CMT-3 at 70 µg/ml for 24 h to study inflammation-induced cell response. The monomeric form of α-synuclein was administered at the same concentration. To confirm that a direct action of CMT-3 was not responsible for the possible reduction of inflammation in αS_PFF_:CMT-3-treated cells, 0.5 µM of this drug was added (the concentration of CMT-3 that would be in wells before dialysis) for 2 h before stimulation with αS_PFF_. α-Synuclein stock solutions used contained less than 0.1 endotoxin unit (EU)/mg protein and were prepared in 20 mM HEPES, 150 mM NaCl, pH 7.4. The residual endotoxins were quantified using the *Limulus* amebocyte lysate assay (Thermo Fisher #88282). The experiments were performed twice with n = 5–6/group for each experiment.

### Molecular dynamics simulations of α-synuclein fibrils

The systems were built and equilibrated using the software package HTMD^78^. The coordinates for the α-synuclein fibril were taken from the solid-state NMR model deposited on PDB (PDB ID: 2N0A)^44^. To limit computational cost, and in accordance with previous simulations of the system^79,80^, we modelled the segment 27–100 containing the Greek motif of the hydrophobic core. The N and C terminal residues were capped with ACE and CT3. The ligand structure was taken from the ChemSpider database^81^ (ChemSpider ID: 28530521) and distributed randomly in the simulation box around the fibril at a minimal distance of 3 Å. The system was solvated using a TIP3P water box and neutralized with 150 mM NaCl for a total of approximately 80 K atoms. Parameters for the protein, solvent and ions were taken from the CHARMm 36 forcefield^82^. Parameters for the ligand were adapted from the CHARMm forcefield for tetracyclines and its analogs developed by Aleksandrov and Simonson^83,84^. Twelve different systems were built and equilibrated independently.

The systems were energy minimized for 1000 steps followed by 5 ns equilibration with a 10 kcal mol^−1^ Å^−2^ harmonical constraint on C-α and 5 ns equilibration without constraints under CPT conditions. Pressure and temperature were set at 1 atm and 300 K using a Berendsen thermostat and barostat, respectively. The systems were simulated under periodic boundary conditions with a 9 Å cutoff for nonbonded interactions and a Particle Mesh Ewald algorithm was applied for the treatment of long-range electrostatic interactions. The integration timestep was set to 4 fs using the hydrogen mass repartition scheme implemented in AceMD^85^.

Production simulations were performed in the NVT ensemble using Amber18 program^86^ with the target temperature controlled via Langevin dynamics^87^. Bonds involving hydrogen atoms were handled through the SHAKE algorithm^88^, allowing for an integration timestep of 2 fs. Each system was simulated for 600 ns and the trajectories fragmented into 100 ns segments. In order to improve sampling of the system, new 100 ns simulations were run from relevant frames identified in the MSM model, a technique known as respawning^89^. Model building and respawning simulations were performed until the Markov model was converged.

### Simulation data analysis

For the analysis of the trajectories we used the pyEMMA package^90^ as implemented in the HTMD software package which allows for the construction of a Markov state model (MSM) for the protein–ligand binding process. Conformationally relevant states of the model were built by projecting the simulation on a lower dimensional metric space such as the protein–ligand contact map. A contact is defined whenever a heavy atom of the ligand is within a minimum distance of 5 Å from the carbon alphas of the protein. The data was further projected into the 3 slowest order parameters using the time-lagged independent component analysis (TICA) with a 20 ns lag time and separated into 1000 cluster using the mini-batch k-means clustering algorithm^91^. The MSM was built using a time-lag of 25 ns and the microstates were lumped into 5 macrostates using the PCCA algorithm^92^. Error estimation was performed by conducting the analysis in several independent runs, where each run was bootstrapped leaving 20% of the dataset out randomly.

### Statistical analyses

All data was obtained from at least three independent experiments and expressed as mean ± SEM. Multiple-group comparisons were performed with one-way ANOVA. Differences were considered as statistically significant at p < 0.05. Statistical analyses were carried out with GraphPad Prism 5 (San Diego, California, USA).

## Supplementary information

Supplementary Information.
