# Supplementary material for: CMT-3 targets different α-synuclein aggregates mitigating their toxic and inflammogenic effects
Source: Sci Rep. 2020 Nov 20;10:20258. doi: 10.1038/s41598-020-76927-0 (PMC7679368; doi:10.1038/s41598-020-76927-0)
Supplement: Supplementary file 1 — Supplementary Information. [file 41598_2020_76927_MOESM1_ESM.docx]

**Supplementary data**

**CMT-3 targets different α-synuclein aggregates mitigating their toxic and inflammogenic effects**

Florencia González-Lizárraga^1^, Diego Ploper^1^, César L. Ávila^1^, Sergio B. Socías^1^, Mauricio dos-Santos-Pereira^2^, Belén Machín^1^, Elaine del Bel^2^, Patrick Pierre Michel^4^, Lía I. Pietrasanta^3^, Rita Raisman-Vozari^4*^ and Rosana Chehín^1*^

^1^Instituto de Investigación en Medicina Molecular y Celular Aplicada (IMMCA) (CONICET-UNT-SIPROSA), Pasaje Dorrego 1080, San Miguel de Tucumán, 4000, Argentina

^2^Faculdade de Odontología de Ribeirão Preto, Universidade de São Paulo, Brazil

^3^Departamento de Física - Instituto de Física de Buenos Aires (IFIBA-CONICET) and Centro de Microscopías Avanzadas (CMA), Facultad de Ciencias Exactas y Naturales, Universidad de Buenos Aires, C1428EHA Buenos Aires, Argentina

^4^ Paris Brain Institute, Inserm U 1127, CNRS UMR 7225, Sorbonne Université, Paris, France

^*^Correspondence must be addressed to RCH (rosanachehin@gmail.com) or RRV (ritaraisman@gmail.com)

Keywords: Parkinson’s disease; chemically modified tetracycline 3; α-synuclein toxicity; neuroinflammation; binding mechanism.

**Supplementary tables, figures and legends**

**Supplementary Table S1.** Band position and percentage area corresponding to the components of Amide I' band of the oligomeric species of α-synuclein in the absence or in the presence of CMT-3 obtained after curve fitting procedure.

|  | Band position  (cm^-1^) | % Area | Assignment |
| --- | --- | --- | --- |
| α-Synuclein  (16h) | 1609  1624  1641  1650  1658  1672  1687 | <1  13.8  32.4  <1  30.8  20.1  2.6 | Side chains  Antiparallel β-Sheet  Disorder/Extended  α-Helix  Loops/Disorder  β-turn  β-Sheet (High component) |
| α-Synuclein:CMT-3  (16h) | 1615  1633  1641  1652  1660  1671  1684 | 4,3  31,4  9,7  23.8  19,1  11  <1 | Extended/Side chains  β-Sheet  Disorder/Extended  α-Helix  Loops/Disorder  β-turn  - |


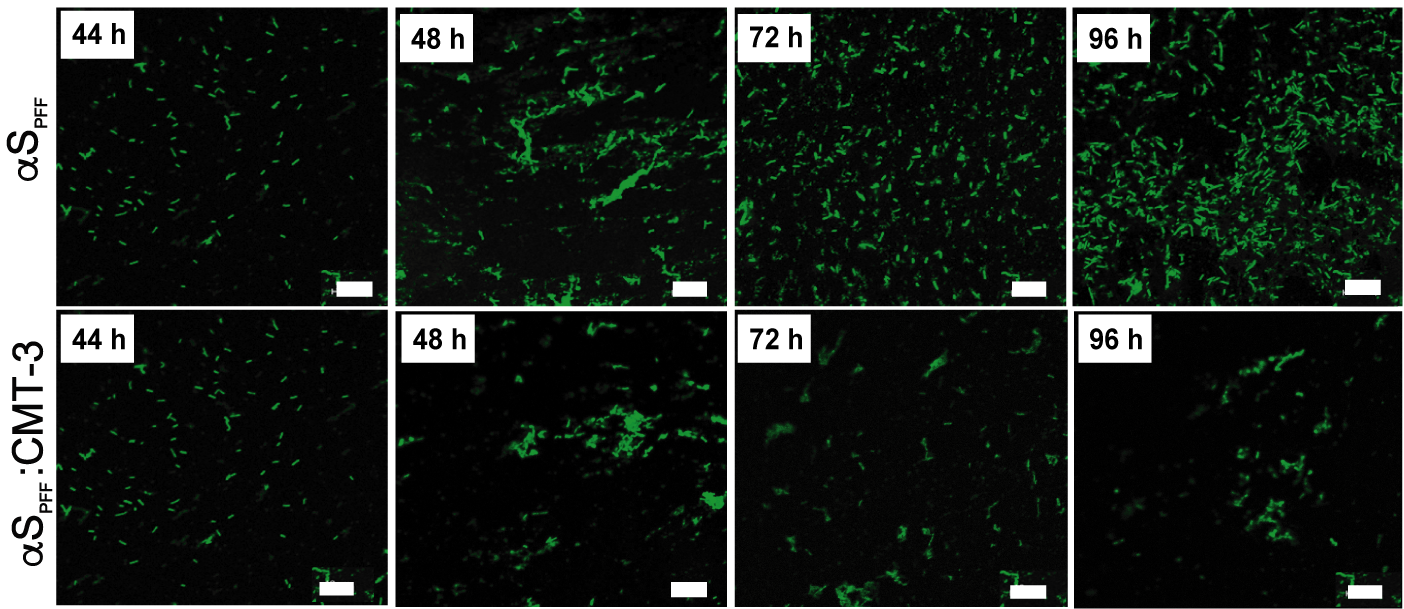


**Supplementary Figure S1. Disruption of α-synuclein amyloid preformed fibrils induced by CMT-3.** Fluorescence confocal microscopy shows the progression of α-synuclein fibril formation upon the addition at 44 h of buffer (top) or CMT-3 (bottom) to samples containing αS_PFF_. Samples were incubated with ThS for 2 h and stirred at room temperature. The scale bar corresponds to 10 μm.


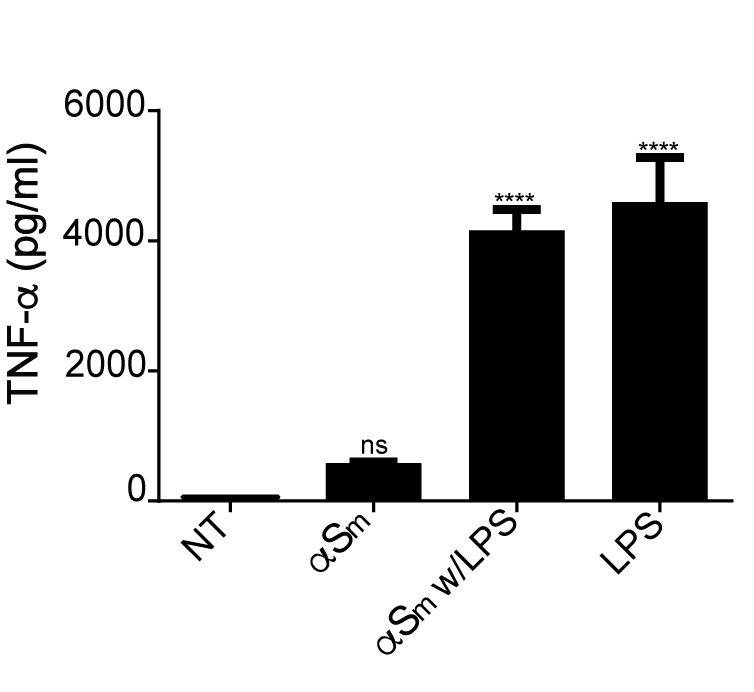


**Supplementary Figure S2. Impact of a 24-hr treatment with α-synuclein monomer (70 μg/ml) on the release of TNF-α in microglial cell cultures.** Comparison of α-synuclein monomer without endotoxin (αS_m_), with endotoxin (αS_m_ w/LPS) and a treatment with the bacterial inflammogen LPS alone (10 ng/ml). Data represents the mean ± S.E.M (n = 11). One-way ANOVA followed by Holm-Sidak’s multiple comparisons test. **** p < 0.001 *vs* NT.


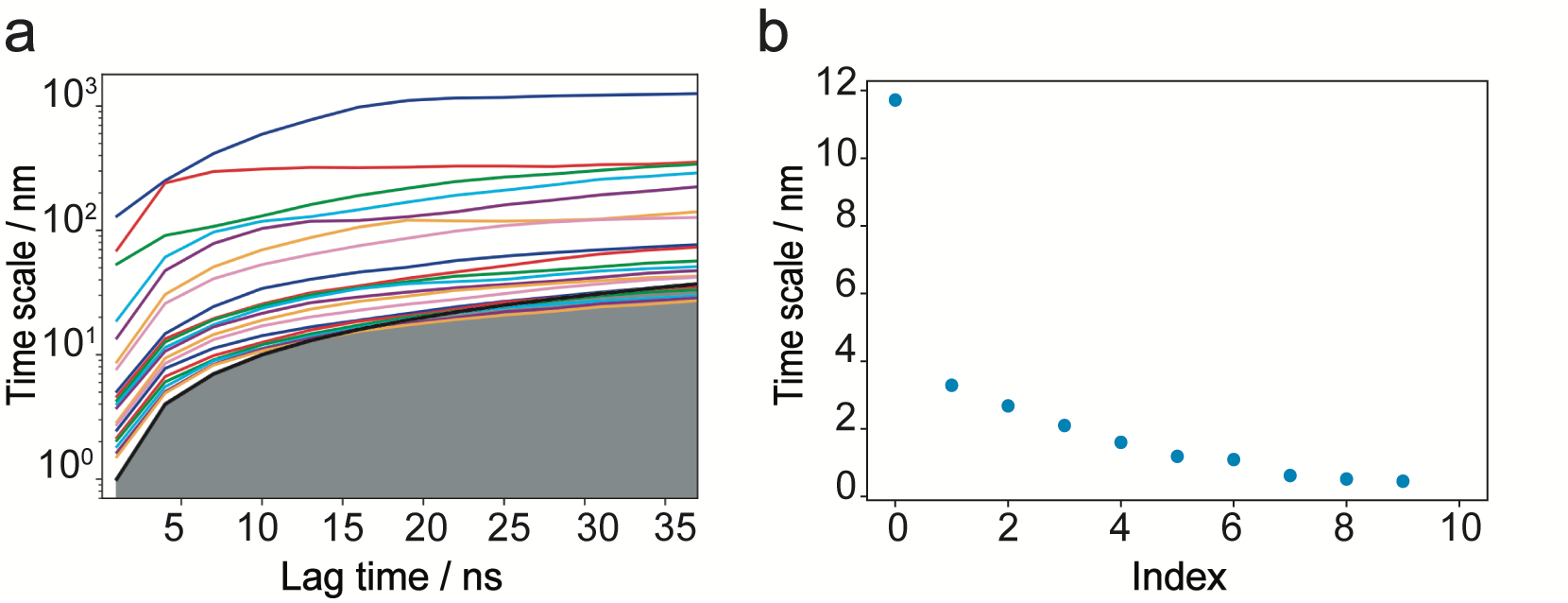


**Supplementary Figure S3. The binding mode of CMT-3 to α-synuclein by high throughput unbiased molecular dynamic simulations.** (**a**) Convergence of the implied time-scale as a function of the lag-time used for the construction of the Markov State Model. (**b**) Implied time scale of the ten slowest process at a lag-time of 20 ns.


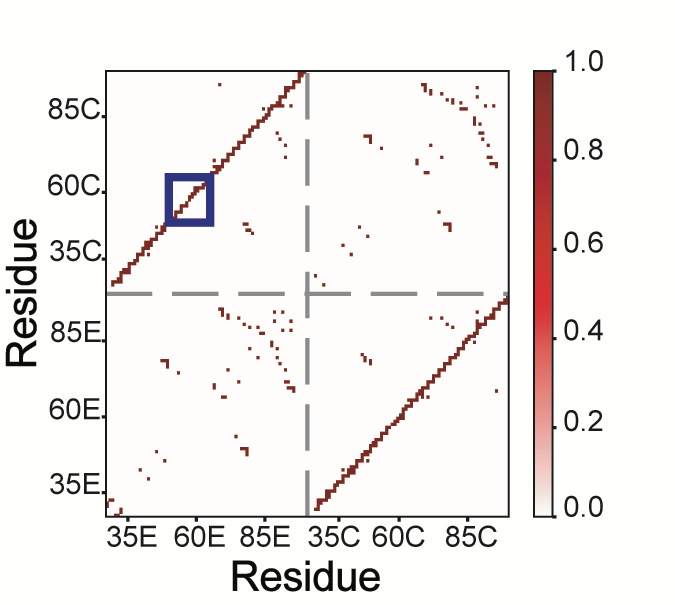


**Supplementary Figure S4. Analysis of contact map.** Contact map between the residues at the edge (E) or core (C) of α-synuclein fiber in the ssNMR structure deposited on PDB (PDBID: 2N0A).


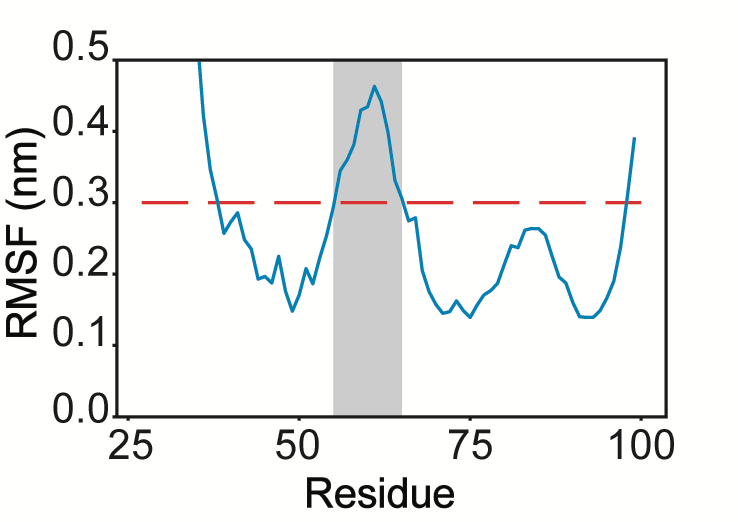


**Supplementary Figure S5. Root mean square fluctuation plot of C-alpha in α-synuclein fibers.** The gray box highlights the flexible loop within the conserved hydrophobic core.
